# ARHGAP11A Is a Prognostic Biomarker and Correlated With Immune Infiltrates in Gastric Cancer

**DOI:** 10.3389/fmolb.2021.720645

**Published:** 2021-10-18

**Authors:** Biao Fan, Ke Ji, Zhaode Bu, Ji Zhang, Heli Yang, Jialin Li, Xiaojiang Wu

**Affiliations:** Key Laboratory of Carcinogenesis and Translational Research (Ministry of Education), Gastrointestinal Cancer Center, Peking University Cancer Hospital and Institute, Beijing, China

**Keywords:** ARHGAP11A, immune infiltrates, gastric cancer, prognosis, biomarker

## Abstract

**Background:** ARHGAP11A, belongs to RhoGAPs family, is vital for cell motility. However, the role of ARHGAP11A in gastric cancer is obscure.

**Methods:** The expression level of ARHGAP11A was analyzed by Oncomine database. The correlation of ARHGAP11A expression with immune infiltrates and associated gene markers was clarified by Tumor IMmune Estimation Resource and Gene Expression Profiling Interactive Analysis database. The correlation between ARHGAP11A expression and the patient prognosis was identified by Kaplan-Meier plotter and PrognoScan. Genetic changes of ARHGAP11A were analyzed by cBioPortal. The protein-protein interaction network and gene functional enrichment analysis were constructed and performed by GeneMANIA and Metascape.

**Results:** We found that the expression levels of ARHGAP11A were elevated in various cancers including gastric cancer when compared with normal tissues. High expression of ARHGAP11A was significantly correlated with a better prognosis in gastric cancer. We revealed that the expression of ARHGAP11A was negatively associated with infiltration levels of CD8^+^ T cells, CD4^+^ T cells, macrophages and dendritic cells. In addition, ARHGAP11A expression was significantly correlated with gene markers of these immune cells. Lastly, gene functional enrichment analysis indicated that ARHGAP11A involved in regulating lymphocyte activation, cell division, cell killing, myeloid leukocyte differentiation and leukocyte apoptosis.

**Conclusion:** Our findings demonstrated that ARHGAP11A was a valuable prognostic biomarker in gastric cancer. Further work is needed to validate its role and underlying mechanisms in regulating immune infiltrates.

## Introduction

Gastric cancer is a common cancer, especially in Asian countries (Sun et al., 2020; Yang et al., 2020; Cao et al., 2021). It is the fourth leading cause of cancer-related mortality worldwide (Sung et al., 2021). In recent years, the immunotherapy has bought revolutionary changes to the treatment of cancer (Ribas and Wolchok, 2018). However, the progress of immunotherapy in gastric cancer was hampered by a limited understanding of the immune microenvironment (Balkwill et al., 2012). The tumor immune microenvironment is crucial for tumor initiation and progression (Salmon et al., 2019). It is composed of all populations of tumor infiltrating cells including macrophages, T cells and dendritic cells (Bruni et al., 2020). Studies have shown that the tumor immune infiltrates correlated with prognosis and response to therapy (Zeng et al., 2019; Refolo et al., 2020). Therefore, there is a clear need to investigate the immune infiltrates related markers and to reveal the molecular mechanisms in gastric cancer.

Rho GTPases are a subfamily of the Ras superfamily proteins which play central roles in multiple biological processes, such as cell motility, cell polarity, cell cycle progression, cell adhesion, migration and invasion. Rho GTPase-activating proteins (RhoGAPs), upstream regulators of Rho GTPases, are frequently dysregulated in various cancers (Porter et al., 2016; Muller et al., 2020). Previous studies showed that Rho GTPases played a role in immune homeostasis, which involved in key processes for the T lymphocytes activation and differentiation (Saoudi et al., 2014). Tumors with abundant tumor-infiltrating lymphocytes (TILs) are associated with a better prognosis than tumors with scarce TILs in various cancers (Brambilla et al., 2016; Sudo et al., 2017). Low-grade TILs are associated with lymph node metastasis of early-stage cancer cells (Zhao et al., 2020). Whether RhoGAPs involve in the tumor immune microenvironment is still unknown. ARHGAP11A, a protein coding gene locates on chr15q13.3, encodes a member of RhoGAPs (ARHGAP11A). The role of ARHGAP11A in cancer is still controversial. In human glioma cells, ARHGAP11A binds to p53 and promotes its function eventually leading to cell-cycle arrest and apoptosis (Xu et al., 2013). ARHGAP11A is upregulated in liver cancer and proceeds the liver cell proliferation and migration via Rac1B (Dai et al., 2018). ARHGAP11A was found involved in the cell migration of breast cancer (Lawson et al., 2016). In our ongoing parallel study, using whole exon and whole genome sequencing, we characterized multiple metastases arising from gastric cancer in twelve patients. We found that high expression of ARHGAP11A, a representative gene with mutation characteristics in the clonal evolution of gastric cancer metastasis, appeared more frequently in gastric cancer with lymph node metastasis (unpublished data). ARHGAP11A might play a key role in lymph node metastasis of gastric cancer. Nevertheless, the prognostic significance of ARHGAP11A and its correlation with immune infiltrates including TILs in gastric cancer is obscure.

In this study, we analyzed the correlation between ARHGAP11A expression and patient prognosis using PrognoScan and Kaplan-Meier plotter. We next investigated the correlation of ARHGAP11A expression and tumor infiltrates in Tumor IMmune Estimation Resource (TIMER) and Gene Expression Profiling Interactive Analysis (GEPIA).

## Materials and Methods

### Oncomine Database Analysis

Oncomine (https://www.oncomine.org/resource/login.html) is a publicly available tumor microarray database and data mining platform that includes 715 datasets and 86,733 samples (includes tumor and normal tissue samples). Gene expression analyses for a single gene can be performed across various types of cancer and include comparisons relative to normal control (Rhodes et al., 2007). The expression of ARHGAP11A in different cancer tissues were analyzed by using Oncomine. The threshold was set as: *p* < 1.0E−04, fold change >2, gene rank: top 10% and data type: mRNA. One dataset (Cui Gastric Dataset: 80 gastric carcinoma and 80 normal paired gastric tissue samples were analyzed (Cui et al., 2011)) met the threshold when compared the ARHGAP11A expression in gastric cancer and normal tissue.

### TIMER Database and GEPIA Database Analysis

TIMER is a public resource for systematic analysis of immune infiltrates across 32 cancer types (10,897 tumor samples) from The Cancer Genome Atlas (TCGA) (https://cistrome.shinyapps.io/timer/) (Li et al., 2017). The DiffExp module of TIMER was used to identify the expression of ARHGAP11A in all TCGA tumors. Gene expression levels were displayed using box plots, with statistical significance of differential expression level of ARHGAP11A evaluated using the Wilcoxon test and marked with asterisk. The Gene module of TIMER database was used to clarify the correlation of ARHGAP11A expression with immune infiltration level, in which the scatterplots was generated and displayed, showed the purity-corrected partial Spearman’s rho value and statistical significance. The Survival module was used to explore the survival differences of patients with different immune infiltrates. The Correlation module of TIMER database was used to draw the expression scatterplots between ARHGAP11A and immune related markers together with the Spearman’s rho value and estimated statistical significance (Correlation adjusted by tumor purity). GEPIA (http://gepia.cancer-pku.cn/index.html) is an online database that includes 9,736 tumors and 8,587 normal samples from TCGA and the GTEx projects (Tang et al., 2017). It was used to further validate the significantly correlated genes in TIMER. Gene expression correlation analysis was performed for given sets of TCGA expression data. The correlation coefficient was determined by the Spearman method.

### Kaplan-Meier Plotter and PrognoScan Analysis

The Kaplan Meier plotter (https://kmplot.com/analysis/) is an online database capable to assess the effect of 54,675 genes (mRNA, miRNA, protein) on survival in 21 cancer types including gastric cancer (1,440 samples) (Szasz et al., 2016). Sources for the databases include GEO, EGA, and TCGA. It was used to analyze the relationship of ARHGAP11A expression with Overall Survival (OS) and Relapse Free Survival (RFS) in various cancer types, and hazard ratio (HR) values with 95% confidence intervals and log-rank *p*-values were calculated. Adjusted analyses were performed by using data from gastric cancer patients with different clinical parameters such as gender, tumor stage, Lauren classification, differentiation, treatment and HER2 status. PrognoScan (http://dna00.bio.kyutech.ac.jp/PrognoScan/index.html) provides a powerful platform to explore the relationships between gene expression and patient prognosis across a large collection of publicly available cancer microarray datasets (Mizuno et al., 2009). The threshold was set as: Corrected *p*-value and Cox *p*-value both <0.05. The impact of both ARHGAP11A expression level and clinical parameters was analyzed.

### cBioPortal Analysis

The cBio Cancer Genomics Portal (https://cbioportal.org) has multidimensional cancer genomics datasets (Gao et al., 2013). Data from 1,120 patients (TCGA datasets) was selected to analyze genetic changes of gastric cancer by using cBioPortal. Genetic alterations were shown in different colors.

### GeneMANIA protein-protein interaction (PPI) Analysis and Metascape Gene Enrichment Analysis

GeneMANIA (http://genemania.org/) is an online tool uses bioinformatic methods to display a list of interacting genes, including gene co-expression, physical interactions, gene co-localization, gene enrichment analysis and website prediction. It always be used to construct a PPI network and analyze the function of interactive genes (Warde-Farley et al., 2010). Metascape (https://metascape.org/gp/index.html) is a gene function annotation website (Zhou et al., 2019). It integrates multiple authoritative data resources such as Gene Ontology (GO), Kyoto Encyclopedia of Genes and Genomes (KEGG) pathway, UniProt and DrugBank to complete thorough pathway enrichment and biological process annotation. A PPI network which contained genes interacting with ARHGAP11A was constructed by using GeneMANIA. Genes identified by the GeneMANIA PPI network and TIMER analysis were included in the GO function analyses by using Metascape.

### Statistical Analysis

Patient survival plots generated from the TIMER, GEPIA, PrognoScan and Kaplan-Meier plotter were displayed with HR, *p* or Cox *p*-value from a log-rank test. *p* < 0.05 was considered statistically significant.

## Results

### Pan-Cancer Analysis of ARHGAP11A Expression Levels

**FIGURE 1:**
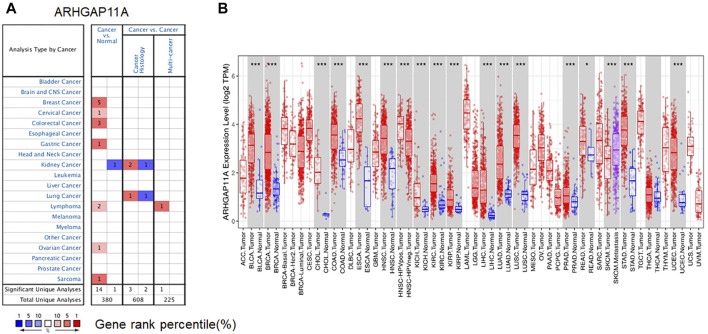
ARHGAP11A expression in different tumor and normal tissues. **(A)** Oncomine. (*p*-value < 1.0E−04), Cell color is determined by the best gene rank percentile for the analyses within the cell. An analysis may be counted in more than one cancer type. **(B)** TIMER. (*p-value < 0.05, *** *p*-value < 1.0E−03).

We firstly analyzed the expression of ARHGAP11A in different tissues by using Oncomine. We revealed that expression levels of ARHGAP11A were elevated in breast, cervical, colorectal, gastric, ovarian cancers, lymphoma and sarcoma relative to normal tissues. In contrast, the ARHGAP11A expression was lower in kidney cancer when compared with normal kidney tissue (Figure 1A). Details were shown in Supplementary Table S1. Next, we assessed the transcriptional levels of ARHGAP11A by using RNA-sequencing data in TCGA and TIMER. Results showed significant differences in ARHGAP11A expression levels when compared tumor and normal tissues (Figure 1B). For example, the transcriptional expression of ARHGAP11A was significantly elevated relative to normal tissues in esophageal carcinoma (ESCA) and stomach adenocarcinoma (STAD). Moreover, the transcriptional expression level of ARHGAP11A in metastatic lesion of skin cutaneous melanoma (SKCM. Metastasis) was significantly higher than in the primary lesion of skin cutaneous melanoma (SKCM. Tumor). In consistent with high transcription level of ARHGAP11A in gastric cancer tissues shown in Figure 1; Cytoplasmic expression of ARHGAP11A was also detected in most cancers including gastric cancer. As shown in Supplementary Figure S1 7 of 9 gastric cancer patients show high/median expression (data from The Human Protein Atlas: https://www.proteinatlas.org/).

### The Prognostic Value of ARHGAP11A Expression in Cancer Patients

**FIGURE 2:**
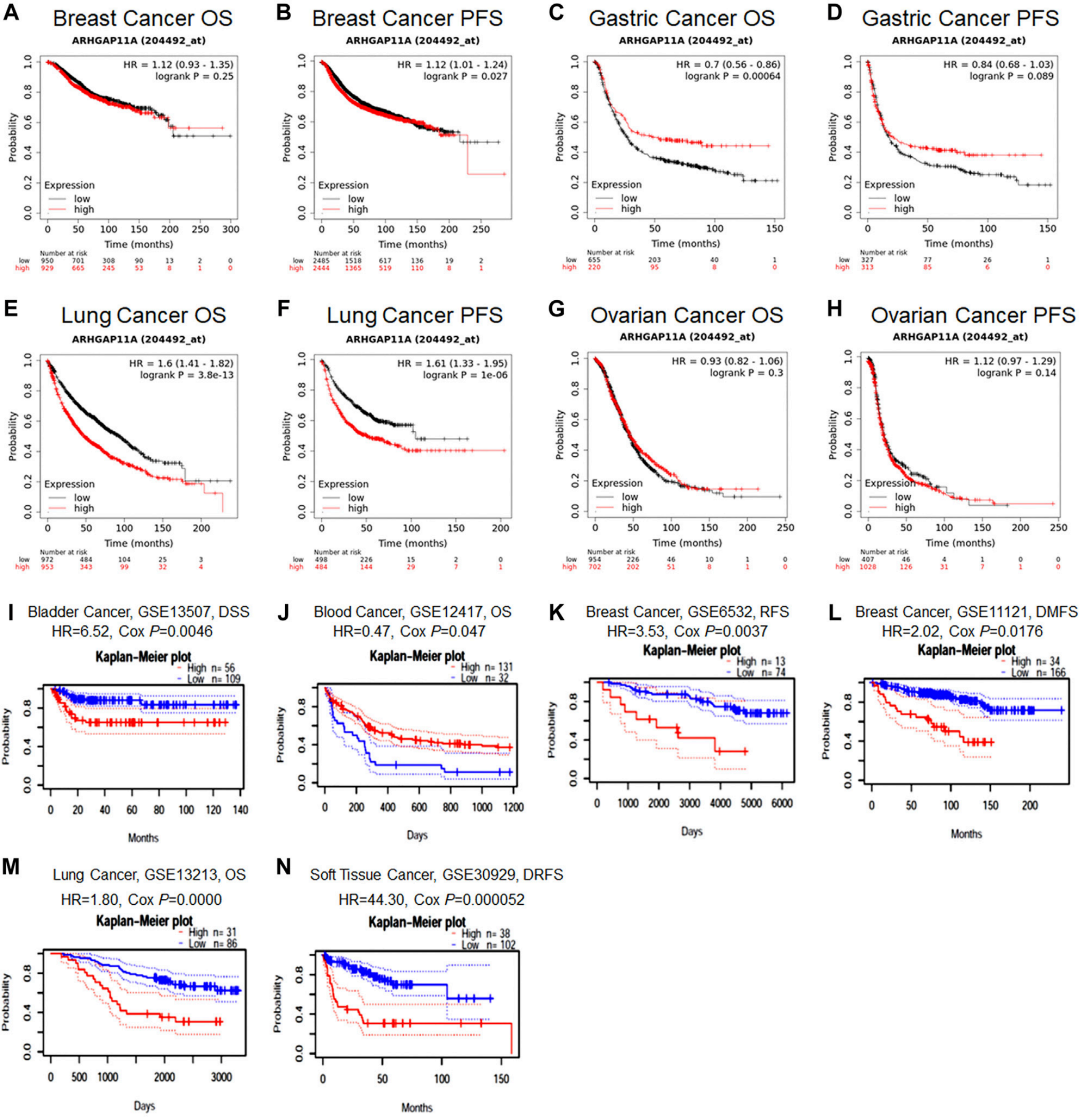
The prognostic value of ARHGAP11A expression in various types of cancer. **(A–H)** Kaplan-Meier Plotter. **(I–N)** PrognoScan. The cohort GSE12417: expression profiling by array; cancer subtype: acute myeloid leukemia; n = 163; endpoint: overall survival. The cohort GSE13213: expression profiling by array; cancer subtype: lung adenocarcinoma; n = 117; patient age: 32–84 years old; endpoint: overall survival. The cohort GSE13507: expression profiling by array; cancer subtype: bladder transitional cell carcinoma; n = 165; patient age: 27–88 years old; sample type: frozen tissue; endpoint: disease specific survival. The cohort GSE6532: expression profiling by array; cancer subtype: breast cancer; n = 87; endpoint: distant metastasis free survival. The cohort GSE11121: expression profiling by array; cancer subtype: breast cancer; n = 200; patient age: 34–89 years old; sample type: frozen; endpoint: distant metastasis free survival. The cohort GSE30929: expression profiling by array; cancer subtype: liposarcoma; n = 140; endpoint: distant recurrence free survival.

We next identified the prognostic value of ARHGAP11A in various cancers by using the Prognoscan and Kaplan-Meier plotter (Figure 2 and Supplementary Tables 2–6). Results revealed that high expression of ARHGAP11A was significantly associated with a better prognosis in gastric cancer (OS HR = 0.7, *p* = 6.4e−04) (Figure 2C) and blood cancer (The cohort GSE12417, OS HR = 0.47, Cox *p* = 0.047) (Figure 2J). In contrast, high expression of ARHGAP11A was correlated with poor prognosis in lung cancer (OS HR = 1.6, *p* = 3.8e−13; Progression free survival (PFS) HR = 1.61, *P* = 1e−06; The cohort GSE13213, OS HR = 1.80, Cox *p* = 0.0000) (Figures 2E,F, 2M), bladder cancer (The cohort GSE13507, disease Specific Survival (DSS) HR = 6.52, Cox *p* = 0.0046) (Figure 2I), breast cancer (The cohort GSE6532, RFS HR = 3.53, Cox *p* = 0.0037; The cohort GSE11121, Distant Metastasis Free Survival (DMFS) HR = 2.02, Cox *p* = 0.0176) (Figures 2K,L) and soft tissue cancer (The cohort GSE30929, Distant relapse free survival (DRFS) HR = 44.30, Cox *p* = 0.000052) (Figure 2N). No significant relationship was identified between the expression of ARHGAP11A and prognosis of breast and ovarian cancer patients (Figures 2A,B,G,H).

### Correlation of ARHGAP11A Expression and Clinical Parameters of Gastric Cancer

To further understand the role of ARHGAP11A in gastric cancer, we analyzed the correlation between the ARHGAP11A expression and clinical parameters by using the Kaplan-Meier plotter. High expression of ARHGAP11A was significantly correlated with better prognosis in patients with specific clinical parameters (*p* < 0.05) (Table 1). Interestingly, the therapeutic strategy and HER2 status influenced the prognostic value of ARHGAP11A. High expression level of ARHGAP11A was associated with better prognosis when the patient treated with surgery alone, while in contrast, associated with worse prognosis when treated with surgery and 5-Fu based adjuvant chemotherapy (Table 1). Moreover, High expression levels of ARHGAP11A indicated better prognosis in HER2 negative patients, while indicated worse prognosis in HER2 positive patients (Table 1).

**TABLE 1:** Correlation of ARHGAP11A and clinical parameters in gastric cancer from Kaplan-Meier Plotter.

Clinical parameters	OS(n = 881)			PFS (n = 645)		
	N	HR	*p*	N	HR	*p*
Sex						
Female	236	0.5 (0.33–0.74)	0.00053	201	0.52 (0.34–0.81)	0.0028
Male	544	0.76 (0.6–0.97)	0.027	437	0.88 (0.68–1.13)	0.31
Tumor stage						
1	67	0 (0-Inf)	0.0012	60	0 (0-Inf)	0.0054
2	140	0.61 (0.3–1.24)	0.17	131	0.74 (0.41–1.36)	0.33
3	305	0.56 (0.4–0.78)	0.00048	186	0.55 (0.36–0.84)	0.0046
4	148	0.66 (0.44–0.98)	0.038	141	0.78 (0.52–1.16)	0.21
Lauren classification						
Intestinal	320	0.37 (0.25–0.55)	1.7e−07	263	0.56 (0.39–0.82)	0.0026
Diffuse	241	0.61 (0.43–0.86)	0.0046	231	0.66 (0.47–0.94)	0.019
Mixed	32	0.49 (0.14–1.77)	0.27	28	1.37 (0.51–3.72)	0.53
Differentiation						
Poor	165	1.29 (0.85–1.96)	0.23	121	0.78 (0.49–1.23)	0.28
Moderate	67	0.58 (0.29–1.14)	0.11	67	0.61 (0.32–1.16)	0.13
Well	32	1.43 (0.55–3.68)	0.46			
Treatment						
Surgery alone	380	0.5 (0.35–0.72)	9.3e−05	375	0.62 (0.44–0.87)	0.0052
5-Fu based adjuvant Chemotherapy	152	2.09 (1.39–3.15)	0.00029	152	2.06 (1.39–3.06)	0.00026
Other adjuvant Chemotherapy	76	0.59 (0.24–1.49)	0.26	80	1.53 (0.69–3.35)	0.29
HER2 status						
Negative	532	0.56 (0.43–0.72)	8.4e−06	408	0.68 (0.52–0.88)	0.0029
Positive	343	1.47 (1.12–1.93)	0.0054	232	1.69 (1.21–2.38)	0.0021

### Relationships Between ARHGAP11A Expression and Immune Infiltrates in Gastric Cancer

**FIGURE 3:**
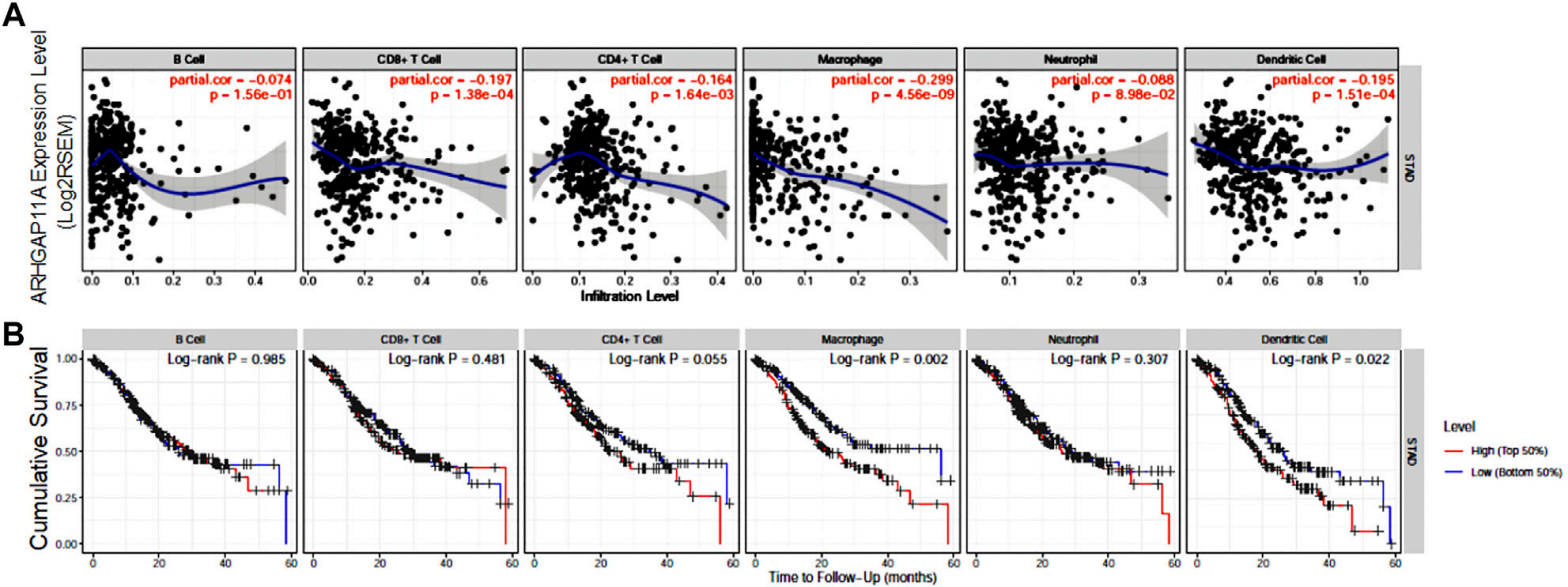
ARHGAP11A expression is correlated with immune infiltrates in gastric cancer. **(A)** Correlation of ARHGAP11A expression with immune cell infiltration. **(B)** Prognostic value of immune cell infiltration in gastric cancer.

We next analyzed the correlation between ARHGAP11A expression and immune infiltrates in gastric cancer by using TIMER (Figure 3). We found that the expression of ARHGAP11A was negatively associated with infiltration levels of CD8^+^ T cells (*p* = 1.38e−04), CD4^+^ T cells (*p* = 1.64e−03), macrophages (*p* = 4.56e−09) and dendritic cells (*p* = 1.51e−04) (Figure 3A). Moreover, the macrophage and dendritic cell infiltration significantly correlate with prognosis of gastric cancer patients in KM survival analysis (Figure 3B). The upper results implied ARHGAP11A might affect patient prognosis via regulating immune infiltrates in gastric cancer.

### Relationships Between ARHGAP11A and Immune Markers Expression

**FIGURE 4:**
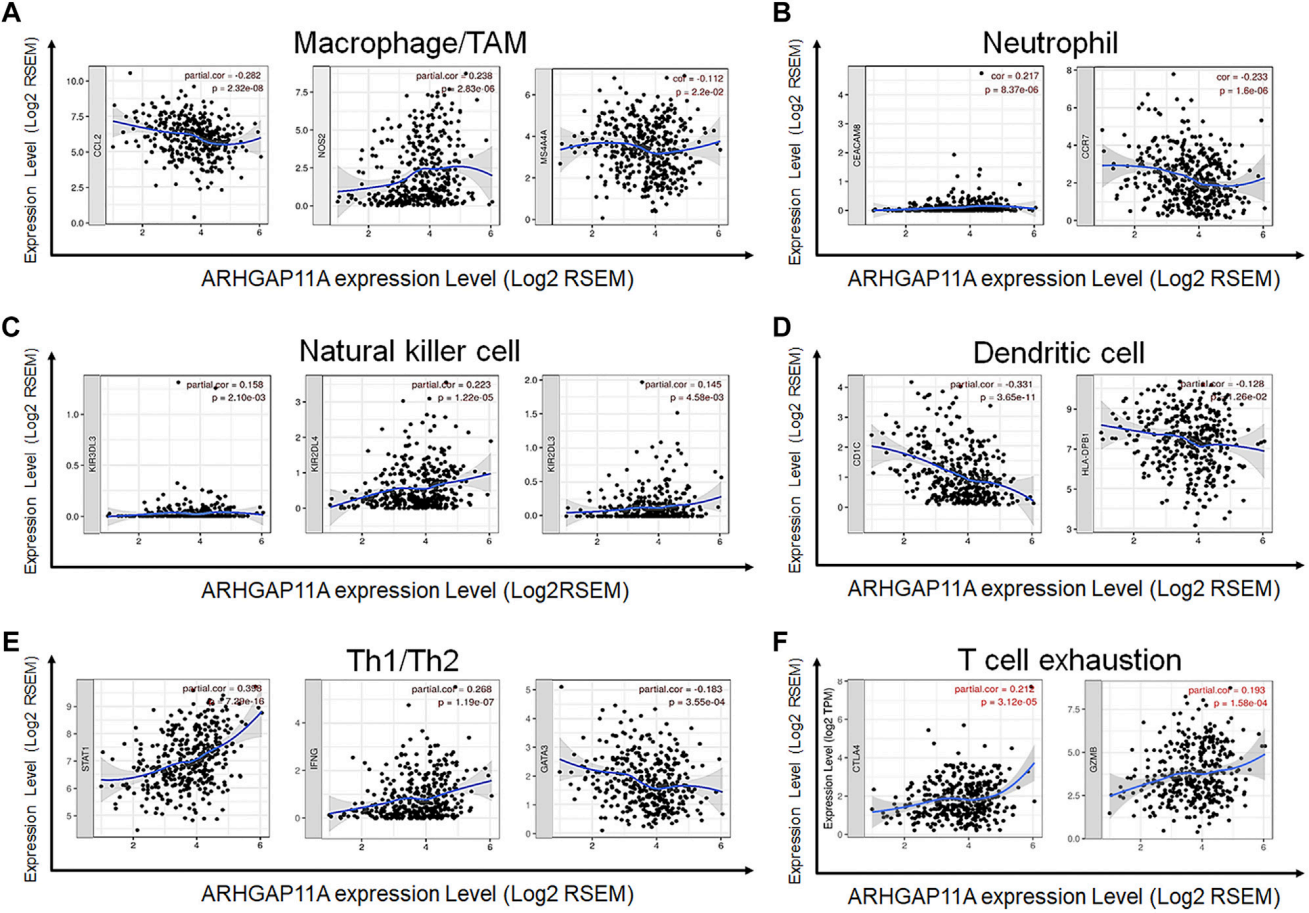
Correlation between ARHGAP11A expression and immune markers in gastric cancer. **(A)** Markers of macrophages/TMAs. **(B)** Markers of neutrophils. **(C)** Markers of nature killer cells. **(D)** Markers of dendritic cells. **(E)** Markers of Th1/Th2 cells. **(F)** Markers of exhausted T cells.

We revealed the correlation between ARHGAP11A expression and gene markers of different types of immune cells by using the TIMER and GEPIA. As shown in Table 2 and Figures 4A–H, ARHGAP11A expression was significantly correlated with multiple immune markers, in particular, including the macrophage/TAM marker (CCL2, NOS2, and MS4A4A), the neutrophil marker (CEACAM8 and CCR7), the natural killer cell marker (KIR2DL3, KIR2DL4, and KIR3DL3), the dendritic cell marker (HLA-DPB1 and CD1C), the Th1/Th2 marker (STAT1, IFNG and GATA3), the exhausted T cell marker (CTLA4 and GZMB). We further evaluated the relationship between ARHGAP11A expression and these immune markers in gastric cancer using the GEPIA. Similar results were shown in Table 3. For example, the M1 Macrophage marker NOS2 and the dendritic cell marker, HLA-DPB1 and CD1C, were significantly correlated with ARHGAP11A expression in gastric cancer. Taken together, ARHGAP11A might involve in infiltration of M1 Macrophages and dendritic cells.

**TABLE 2:** Correlation between ARHGAP11A and related immune markers in TIMER.

Cell types	Gene markers	Gastric cancer			
		None		Purity	
		Correlation	*p*	Correlation	*p*
CD8^+^ T cell	CD8A	−0.0074	0.13	−0.0052	0.317
	CD8B	−0.0023	0.645	0.007	0.891
T cell general	CD3D	−0.0098	*	−0.042	0.415
	CD3E	−0.118	*	−0.064	0.214
	CD2	−0.056	0.252	−0.007	0.892
B cell	CD19	−0.164	***	−0.142	**
	CD79A	−0.252	***	−0.227	***
Monocyte	CD86	−0.02	0.68	0.023	0.655
	CSF1R	−0.112	*	−0.103	*
TAM	CCL2	−0.291	***	−0.282	***
	CD68	0.002	0.962	0.017	0.738
	IL10	−0.007	0.887	0.027	0.604
M1 Macrophage	NOS2	0.22	***	0.238	***
	IRF5	−0.051	0.297	−0.05	0.328
	PTGS2	0.035	0.479	0.046	0.367
M2 Macrophage	CD163	0.057	0.243	0.075	0.143
	VSIG4	−0.108	*	−0.096	0.0652
	MS4A4A	−0.112	*	−0.09	0.0786
Neutrophils	CEACAM8	0.217	***	0.224	***
	ITGAM	−0.064	0.19	−0.049	0.345
	CCR7	−0.233	***	−0.192	***
Natural killer cell	KIR2DL1	0.121	*	0.144	**
	KIR2DL3	0.127	**	0.145	**
	KIR2DL4	0.184	***	0.223	***
	KIR3DL1	0.06	0.224	0.05	0.328
	KIR3DL2	0.109	*	0.132	*
	KIR3DL3	0.155	*	0.158	**
	KIR2DS4	0.092	0.0621	0.108	*
Dendritic cell	HLA-DPB1	−0.169	***	−0.128	*
	HLA-DQB1	−0.083	0.0915	−0.03	0.564
	HLA-DRA	−0.06	0.226	−0.01	0.849
	HLA-DPA1	−0.108	*	−0.065	0.205
	CD1C	−0.347	***	−0.331	***
	NRP1	−0.065	0.187	−0.057	0.265
	ITGAX	0.067	0.172	0.113	*
Th1	TBX21	−0.009	0.85	0.026	0.611
	STAT4	−0.015	0.763	0.021	0.681
	STAT1	0.394	***	0.398	***
	IFNG	0.227	***	0.268	***
	TNF	0.01	0.845	0.045	0.388
Th2	GATA3	−0.207	***	−0.184	***
	STAT6	0.106	*	0.099	0.0548
	STAT5A	0.105	*	0.121	*
	IL13	0.03	0.543	0.047	0.359
Follicular helper T cell	BCL6	−0.171	**	−0.167	**
	IL21	0.161	*	0.2	***
T helper cell	STAT3	0.119	*	0.113	*
	IL17A	0.188	***	0.223	***
Regulatory T cell	FOXP3	0.059	0.228	0.102	*
	CCR8	0.082	0.0967	0.103	*
	STAT5B	0.036	0.466	0.037	0.470
	TGFB1	−0.169	**	−0.162	**
Exhausted T cell	PDCD1	0.011	0.819	0.051	0.325
	CTLA4	0.158	*	0.212	***
	LAG3	0.068	0.164	0.098	0.0561
	HAVCR2	0.061	0.212	0.095	0.0646
	GZMB	0.144	*	0.193	***

Purity: correlation adjusted by purity. *p < 0.05, **p < 0.01, ***p < 0.001.

**TABLE 3:** Correlation between ARHGAP11A and related immune markers in GEPIA.

Cell types	Gene markers	Gastric tissues			
		Tumor		Non-tumor	
		R	*p*	R	*p*
T cell general	CD3E	−0.12	*	0.052	0.76
TAM	CCL2	−0.16	***	−0.37	*
M1 Macrophage	NOS2	0.15	**	0.2	0.24
Neutrophils	CCR7	−0.11	*	−0.035	0.84
Natural killer cell	KIR2DL3	0.114	**	0.140	0.41
	KIR2DL4	0.12	*	0.088	0.61
Dendritic cell	HLA-DPB1	−0.1	*	0.065	0.71
	CD1C	−0.24	***	−0.022	0.9
Th1	STAT1	0.47	***	0.21	0.23
	IFNG	0.13	**	−0.035	0.84
Th2	GATA3	−0.098	*	0.096	0.58
Tfh	IL21	0.11	*	0.011	0.95
T cell exhaustion	CTLA4	0.28	***	−0.028	0.87

*p < 0.05, **p < 0.01, ***p < 0.001.

### Genetic Alteration, PPI Network and Enrichment Analyses of ARHGAP11A

**FIGURE 5:**
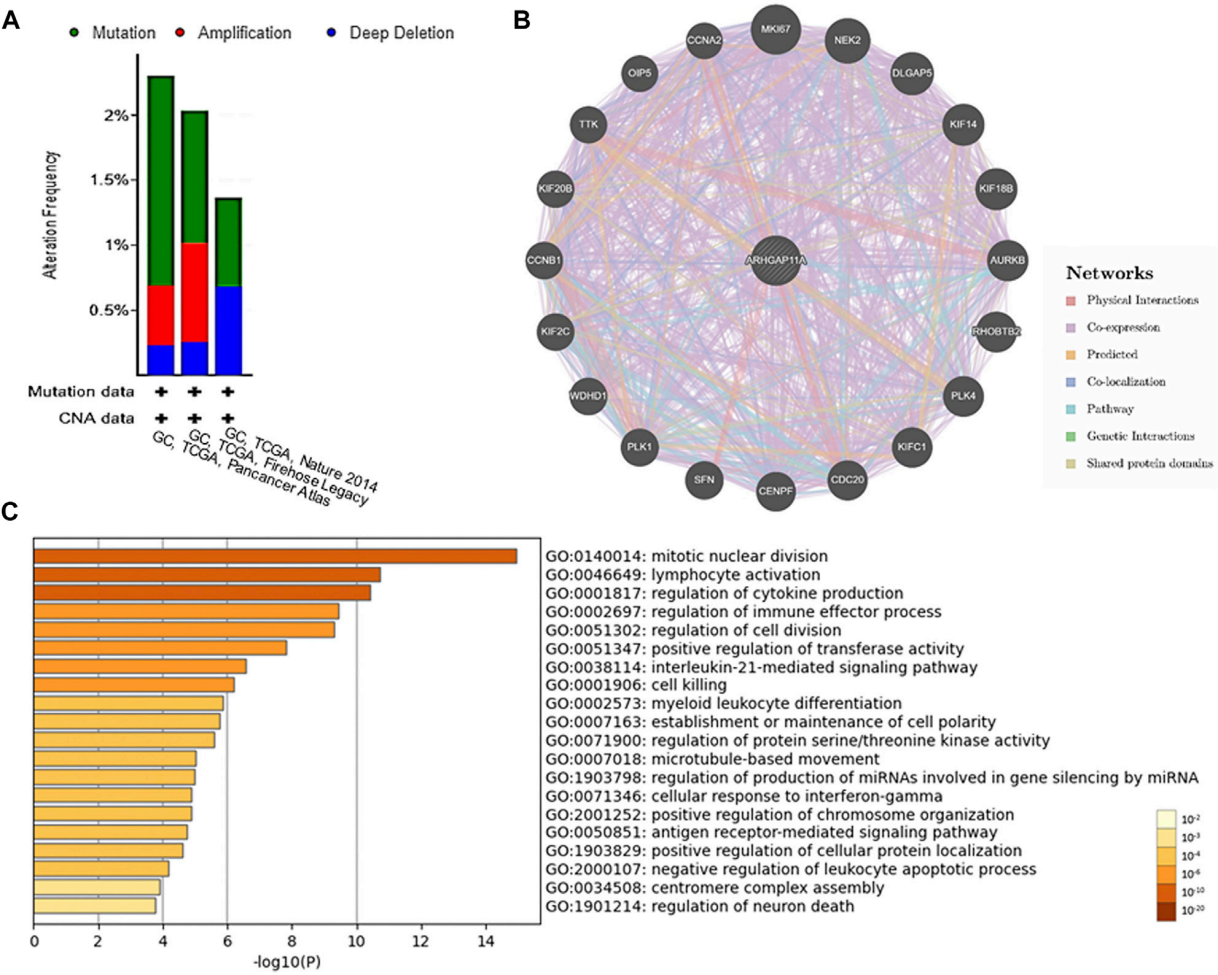
Genetic alteration, PPI network and enrichment analyses of ARHGAP11A. **(A)** Genetic alteration of ARHGAP11A in gastric cancer. **(B)** PPI network of ARHGAP11A in GeneMANIA. **(C)** A heat map of GO function and KEGG pathway analysis of ARHGAP11A and its interacting proteins.

We used the cBioPortal to analyze genetic changes in gastric cancer. Among 1,120 gastric cancer patients, ARHGAP11A was changed in 22 samples (2%), including 13 mutations, 5 amplifications and 4 deep deletions (Figure 5A and Supplementary Figure S2). In addition, ARHGAP11A mutations did not affect the prognosis of gastric cancer (Supplementary Figures S3, 4). The difference of prognosis between ARHGAP11A altered and unaltered group with no statistical significance might due to low frequency of ARHGAP11A alteration. In GeneMANIA analysis, molecular signals interacting with ARHGAP11A included MKI67, MEK2, DLGAP5, KIF14, KIF18B, AURKB, RHOBTB2, PLK4, KIFC1, CDC20, CENPF, SFN, PLK1, WDHD1, KIF2C, CCNB1, KIF20B, TTK, OIP5 and CCNA2 (Figure 5B). Genes from the PPI network and TIMER analysis were included in the GO function and KEGG pathway analyses by using Metascape. Results showed that ARHGAP11A and its interacting signals involved in regulating of lymphocyte activation, immune effector process, cell killing, myeloid leukocyte differentiation, antigen receptor-mediated signaling pathway and leukocyte apoptotic process (Figure 5C).

## Discussion

Dysregulation of Rho GTPases is identified in multiple cancers, and is associated with cancer development and malignant phenotypes. The activity of Rho GTPases (GDP/GTP cycling) is precisely controlled by regulators including GTPase-activating proteins (GAPs) (Lavanderos et al., 2020). Altered expression of GAPs is present in various cancers. ARHGAP10, a member of GAPs, is downregulated in ovarian and breast cancer (Luo et al., 2016; Li et al., 2019). ARHGAP5 is upregulated in metastatic colorectal cancers (Tian et al., 2020). In our study, we systematically analyzed the expression of ARHGAP11A in different tissue types. The expression of ARHGAP11A was found significantly higher in gastrointestinal cancers including stomach adenocarcinoma. Expression level of ARHGAP11A in the metastatic lesion of SKCM was higher than primary SKCM. In addition, the high expression level of ARHGAP11A was associated with better prognosis in gastric and blood cancer, while correlated with poor prognosis in lung, bladder, breast and soft tissue cancer. Taken together, ARHGAP11A revealed dual effects on different human cancers.

Interestingly, an interaction effect exists between ARHGAP11A expression and therapeutic strategy on the prognosis of gastric cancer. High expression level of ARHGAP11A was associated with better prognosis when the patient treated with surgery alone, while in contrast, associated with worse prognosis when treated with surgery and 5-Fu based adjuvant chemotherapy. Microsatellite instability (MSI) is a mature biomarker for predicting the efficacy of immune checkpoint inhibitors. Similar effects were reported when MSI status was applied to predict the chemo-sensitivity in locally advanced colorectal cancer. Efficacy of 5-Fu based adjuvant chemotherapy was significantly different in MSI-H and MSI-L/MSS tumors (Ribic et al., 2003; Klingbiel et al., 2015). We consequently explored the correlation between ARHGAP11A expression and immune infiltrates in gastric cancer.

The lymphocyte in the immune microenvironment is a predictor of sentinel lymph node metastasis and patient survival (Azimi et al., 2012). The evaluation of immune infiltrates in gastric cancer showed that ARHGAP11A expression was significantly associated CD8^+^ T cells, CD4^+^ T cells, macrophages and dendritic cells. Immune genes correlated with ARHGAP11A expression included CCL2, NOS2, CCR7, KIR2DL3, KIR2DL4, HLA-DPB1, CD1C, STAT1, IFNG, and GATA3 and CTLA4. CCL2 is an inflammatory chemokine that promotes the recruitment of Tumor-associated macrophages (TAMs) to tumor sites (Nakatsumi et al., 2017). Chen C, et al. identified a long noncoding RNA, termed Lymph Node Metastasis Associated Transcript 1 (LNMAT1). Mechanistically, LNMAT1 epigenetically activates CCL2 expression and recruits macrophages into bladder cancer, which promotes lymphatic metastasis via VEGF-C excretion (Chen et al., 2018). In our ongoing parallel study, high expression of ARHGAP11A appeared more frequently in gastric cancer with lymph node metastasis. Silencing ARHGAP11A *in vitro*, resulting in the decrease of the invasive ability of gastric cancer cells to lymphatic endothelial cells (unpublished data). ARHGAP11A might play a key role in lymph node metastasis of gastric cancer. Whether LNMAT1 and VEGF-C involves in the biomolecular process needs to be verified in the future study. M1 macrophage marker NOS2 can activate macrophages and causes tumor cell death (Brune et al., 2017). KIR2DL3 and KIR2DL4 are transmembrane glycoproteins expressed by natural killer cells (Gomez-Luque et al., 2021). We found that expression of ARHGAP11A was correlated with NOS2, KIR2DL3 and KIR2DL4. Whether ARHGAP11A involves in regulating macrophages and natural kill cells is worth to be explored. Studies have shown that T cell infiltration defined immune-evasive environment in gastric cancer patients (Gu et al., 2020). In our study, the expression of ARHGAP11A was correlated with multiple T cell markers including STAT1, IFNG, GATA3, and CTLA4. ARHGAP11A might involve in the T cell infiltration of gastric cancer.

Mutations of Rho GTPases have been identified in various cancers. *Rac1* mutation was found in 4–9% of melanomas (Hodis et al., 2012). *RhoA* mutations were identified in over half of angioimmunoblastic T cell lymphomas (Sakata-Yanagimoto et al., 2014). In our study, genetic alterations of *ARHGAP11A* were identified in 2% of gastric cancer. Genes interacting with *ARHGAP11A* included *DLGAP5*, *KIF14*, *AURKB,* and *TTK*. Enhanced expression of DLGAP5 is observed in colorectal cancer. It defines a more aggressive type of colorectal cancer (Branchi et al., 2019). *KIF14* is a potential oncogene, promotes gastric cancer progression and metastasis (Yang et al., 2019). *AURKB* and *TTK* participate in chromosomes segregation during mitosis via regulating kinetochore metaphase signaling (Su et al., 2021). Gene functional enrichment analysis showed ARHGAP11A and its interacting proteins involved in numerous processes including lymphocyte activation, cell division, cell killing, immune effector process regulating, and myeloid leukocyte differentiation.

There are still some limitations in our study. Kaplan Meier estimates are unadjusted in the Prognoscan and the results might be biased. Subgroup analysis is needed to confirm the prognostic value of ARHGAP11A expression in various types of cancer. More *in vivo* and *in vitro* experiments are needed to verify the abovementioned bioinformatic findings, especially the correlation between ARHGAP11A expression and immune infiltrates. On the other hand, the detailed mechanisms of ARHGAP11A in regulating gastric cancer metastasis needs further study.

## Conclusion

In summary, ARHGAP11A might be a crucial regulator of immune infiltrates and a valuable prognostic marker in patients with gastric cancer. Additional studies are needed to validate its role both *in vitro* and *in vivo*.

## Data Availability

The datasets presented in this study can be found in online repositories. The names of the repository/repositories and accession number(s) can be found in the article/Supplementary Material.

## Supplementary Material

Supplementary Figure 1 Cytoplasmic expression of ARHGAP11A in various types of cancer.

Supplementary Figure 2 Genetic alterations of ARHGAP11A in gastric cancer from TCGA database.

Supplementary Figure 3 The overall survival of gastric cancer patients with/without *ARHGAP11A* alteration.

Supplementary Figure 4 The disease-free survival of gastric cancer patients with/without *ARHGAP11A* alteration.

## Glossary

ACC: Adrenocortical carcinoma
BLCA: Bladder urothelial carcinoma
BRCA: Breast invasive carcinoma
CESC: Cervical squamous cell carcinoma and endocervical adenocarcinoma
CHOL: Cholangiocarcinoma
COAD: Colon adenocarcinoma
DLBC: Lymphoid Neoplasm Diffuse Large B-cell Lymphoma
DMFS: Distant Metastasis Free Survival
DRFS: Distant relapse free survival
DSS: Disease Specific Survival
ESCA: Esophageal carcinoma
GAPs: GTPase-activating proteins
GBM: Glioblastoma multiforme
GEPIA: Gene Expression Profiling Interactive Analysis
HNSC: Head and neck squamous cell carcinoma
HR: Hazard ratio
KICH: Kidney chromophobe
KIRC: Kidney renal clear cell carcinoma
KIRP: Kidney renal papillary cell carcinoma
LAML: Acute Myeloid Leukemia
LGG: Brain Lower Grade Glioma
LIHC: Liver hepatocellular carcinoma
LUAD: Lung adenocarcinoma
LUSC: Lung squamous cell carcinoma
MESO: Mesothelioma Rectum adenocarcinoma
MSI: Microsatellite instability
OS: Overall survival
OV: Ovarian serous cystadenocarcinoma
PFS: Progression free survival
PPI: protein-protein interaction
PRAD: Prostate adenocarcinoma
READ: Rectum adenocarcinoma
RFS: Relapse Free Survival
RhoGAPs: Rho GTPase-activating proteins
SARC: Sarcoma
SKCM: Skin cutaneous melanoma
STAD: Stomach adenocarcinoma
TAMs: Tumor-associated macrophages
TCGA: The Cancer Genome Atlas
TGCT: Testicular Germ Cell Tumors
THCA: Thyroid carcinoma
THYM: Thymoma
TILs: tumor-infiltrating lymphocytes
TIMER: Tumor IMmune Estimation Resource
UCEC: Uterine corpus endometrial carcinoma
UCS: Uterine Carcinosarcoma
UVM: Uveal Melanoma
