# Supplementary figures and images for: ARHGAP11A Is a Prognostic Biomarker and Correlated With Immune Infiltrates in Gastric Cancer
Source: Front Mol Biosci. 2021 Oct 18;8:720645. doi: 10.3389/fmolb.2021.720645 (PMC8558302; doi:10.3389/fmolb.2021.720645)

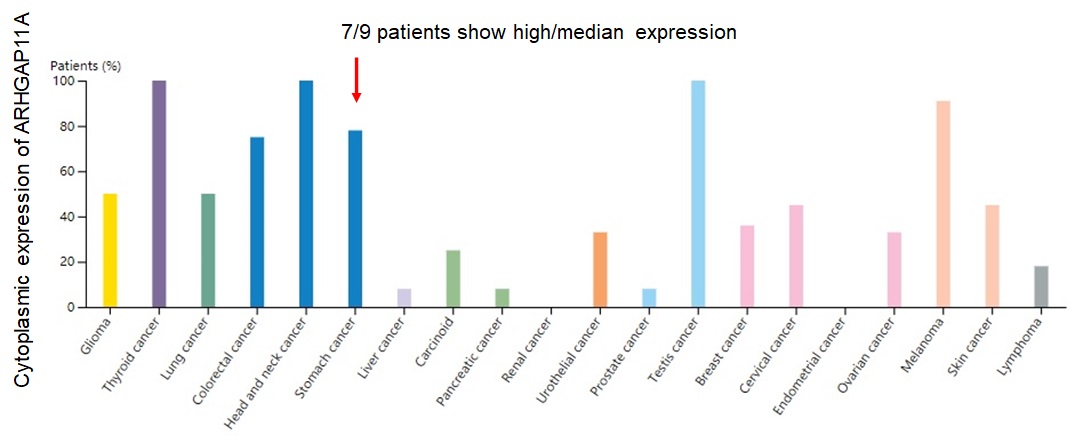

Supplement: Supplementary file 1 [file Image1.JPEG]

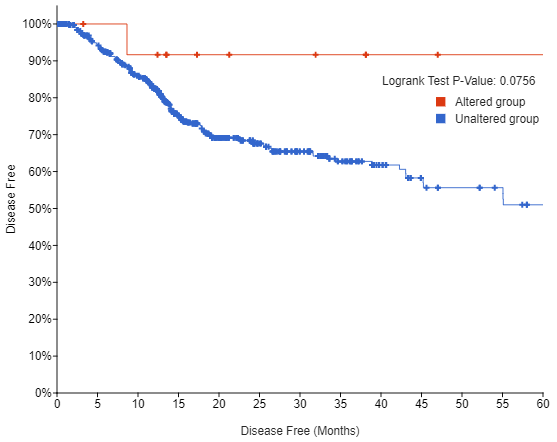

Supplement: Supplementary file 2 [file Image4.PNG]

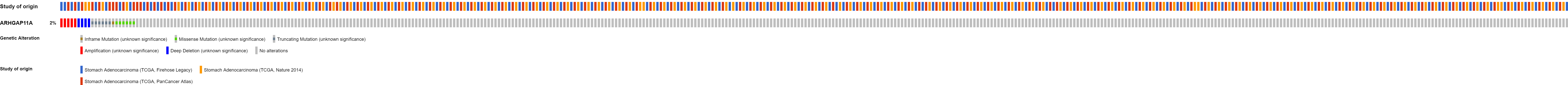

Supplement: Supplementary file 3 [file Image2.PNG]

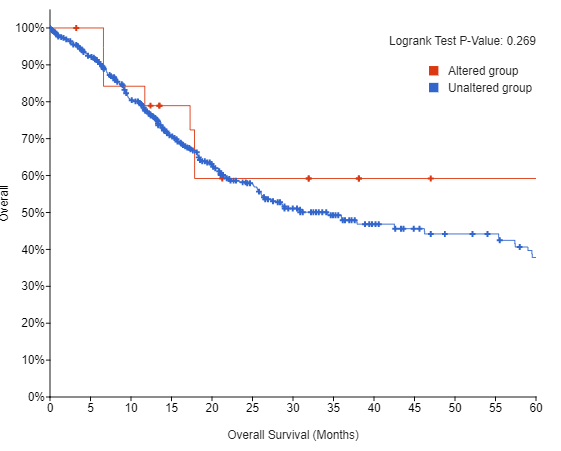

Supplement: Supplementary file 4 [file Image3.PNG]
