# Supplementary material for: ARHGAP11A Is a Prognostic Biomarker and Correlated With Immune Infiltrates in Gastric Cancer
Source: Front Mol Biosci. 2021 Oct 18;8:720645. doi: 10.3389/fmolb.2021.720645 (PMC8558302; doi:10.3389/fmolb.2021.720645)
Supplement: Supplementary file 5 [file DataSheet1.docx]

SUPPLEMENTARY TABLE 1. ARHGAP11A expression in cancers vs normal tissue in oncomine database.

| Cancer | Cancer type | *P*-value | Fold change | Rank (%) | Sample |
| --- | --- | --- | --- | --- | --- |
| Breast | Male Breast Carcinoma | 1.40E-9 | 3.065 | 2% | 64 |
|  | Invasive Ductal Breast Carcinoma | 1.99E-34 | 3.757 | 2% | 450 |
|  | Mucinous Breast Carcinoma | 1.63E-4 | 3.733 | 3% | 65 |
|  | Invasive Lobular Breast Carcinoma | 5.00E-12 | 2.544 | 4% | 97 |
|  | Mixed Lobular and Ductal Breast Carcinoma | 7.94E-5 | 2.679 | 4% | 68 |
|  | Breast Phyllodes Tumor | 3.50E-4 | 1.244 | 1% | 149 |
|  | Ductal Breast Carcinoma in Situ | 7.94E-5 | 1.315 | 3% | 154 |
|  | Invasive Lobular Breast Carcinoma | 2.23E-36 | 1.215 | 3% | 292 |
|  | Mucinous Breast Carcinoma | 9.80E-15 | 1.236 | 4% | 190 |
|  | Tubular Breast Carcinoma | 3.51E-22 | 1.252 | 4% | 211 |
|  | Medullary Breast Carcinoma | 1.21E-10 | 1.379 | 4% | 176 |
| Cervical | Cervical Cancer | 1.57E-7 | 2.154 | 6% | 42 |
| Colorectal | Rectosigmoid Adenocarcinoma | 3.31E-8 | 2.171 | 2% | 25 |
|  | Colon Mucinous Adenocarcinoma | 5.75E-9 | 2.389 | 6% | 44 |
|  | Rectal Adenoma | 9.11E-7 | 2.757 | 3% | 39 |
| Gastric | Gastric Cancer | 6.42E-7 | 2.584 | 2% | 160 |
|  | Diffuse Gastric Adenocarcinoma | 1.60E-4 | 1.174 | 7% | 50 |
|  | Gastric Mixed Adenocarcinoma | 1.00E-3 | 1.141 | 7% | 29 |
|  | Gastric Intestinal Type Adenocarcinoma | 3.00E-3 | 1.112 | 9% | 39 |
| Kidney | Renal Oncocytoma | 6.55E-18 | -2.712 | 2% | 35 |
|  | Papillary Renal Cell Carcinoma | 6.84E-8 | -1.387 | 8% | 34 |
|  | Papillary Renal Cell Carcinoma | 5.83E-4 | -1.095 | 8% | 566 |
| Lymphoma | Unspecified Peripheral T-Cell Lymphoma | 2.46E-9 | 2.036 | 8% | 48 |
|  | Diffuse Large B-Cell Lymphoma | 1.00E-3 | 2.032 | 9% | 51 |
| Ovarian | Ovarian Serous Adenocarcinoma | 5.01E-5 | 5.103 | 8% | 49 |
| Sarcoma | Detwiller Sarcoma | 3.99E-5 | 3.353 | 3% | 22 |
|  | Pleomorphic Liposarcoma | 4.84E-4 | 2.486 | 3% | 18 |
|  | Malignant Fibrous Histiocytoma | 2.62E-4 | 3.087 | 7% | 24 |
|  | Myxofibrosarcoma | 2.92E-8 | 1.217 | 7% | 40 |

SUPPLEMENTARY TABLE 2. Relation between ARHGAP11A expression and patient prognosis of different cancer (Disease Free Survival) in Prognoscan database.


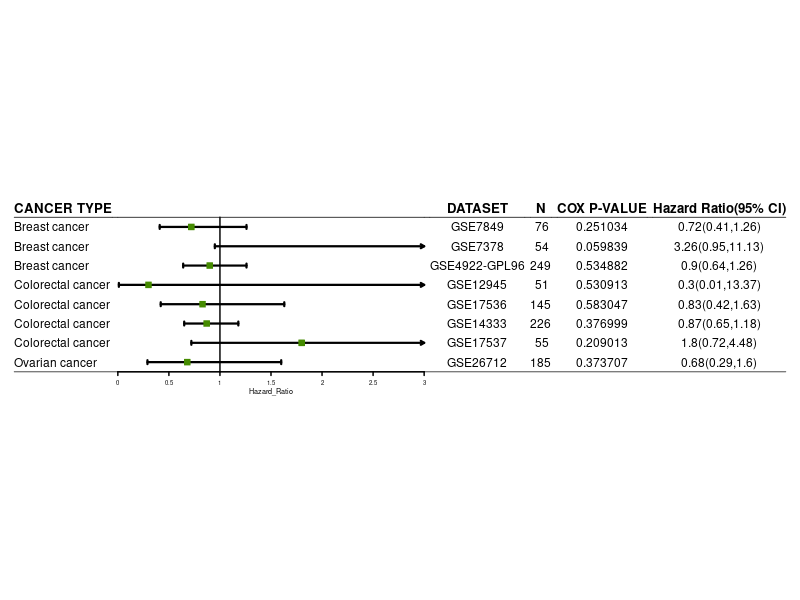


SUPPLEMENTARY TABLE 3. Relation between ARHGAP11A expression and patient prognosis of different cancer (Distant Metastasis Free Survival) in Prognoscan database.

**
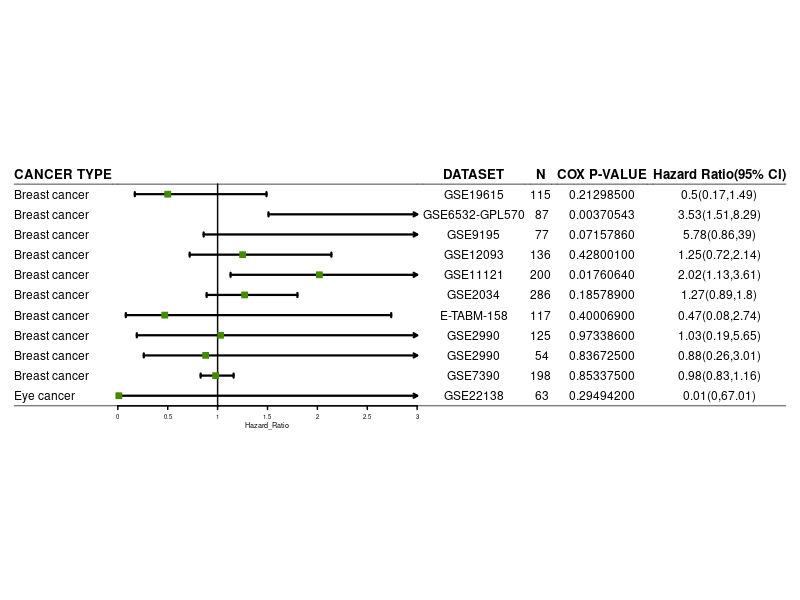
**

SUPPLEMENTARY TABLE 4. Relation between ARHGAP11A expression and patient prognosis of different cancer (Disease Specific Survival) in Prognoscan database.


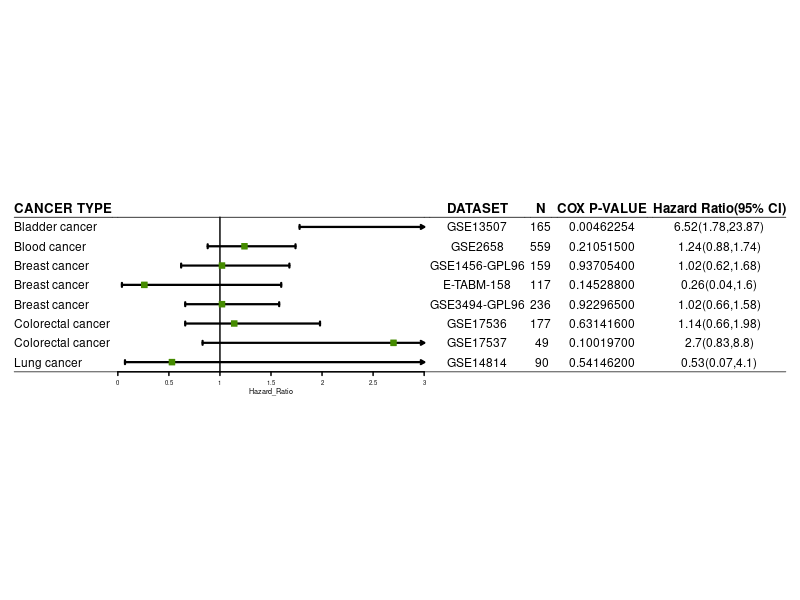


SUPPLEMENTARY TABLE 5. Relation between ARHGAP11A expression and patient prognosis of different cancer (Overall Survival) in Prognoscan database.

**
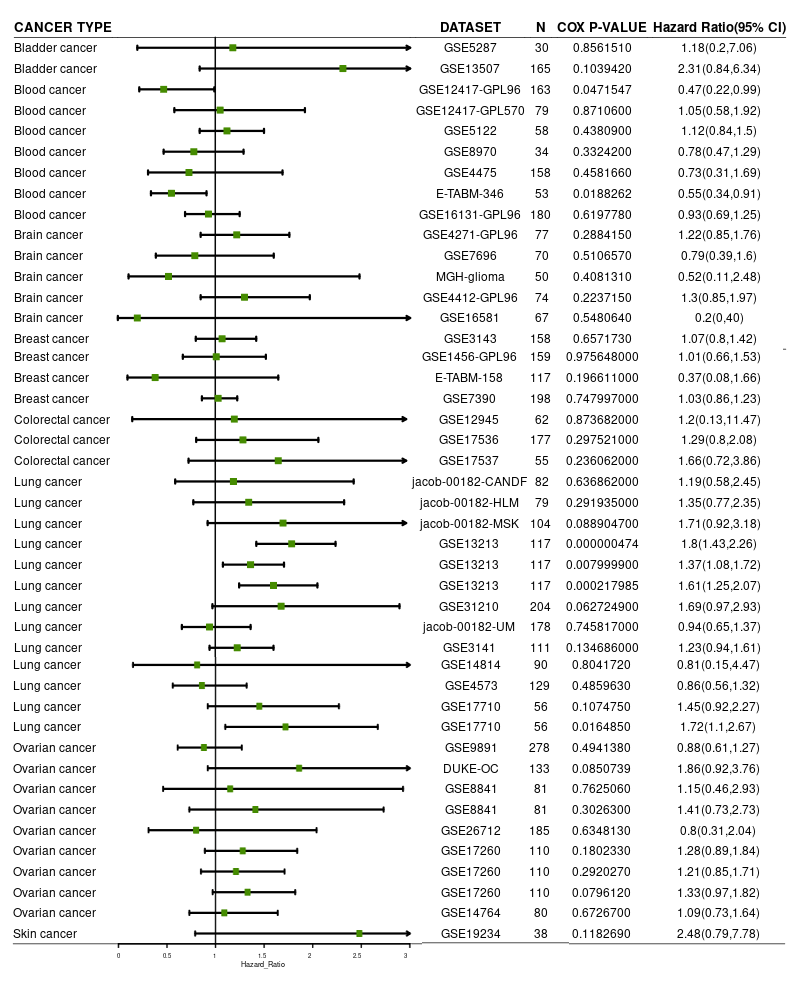
**

SUPPLEMENTARY TABLE 6. Relation between ARHGAP11A expression and patient prognosis of different cancer (Relapse Free Survival) in Prognoscan database.

**
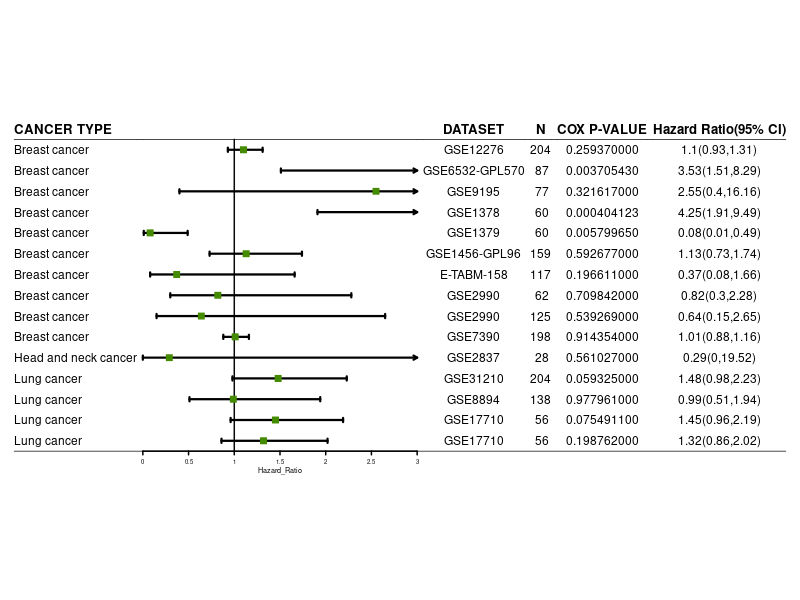
**
